# Supplementary material for: Optical control of PIEZO1 channels
Source: Nat Commun. 2023 Mar 7;14:1269. doi: 10.1038/s41467-023-36931-0 (PMC9992513; doi:10.1038/s41467-023-36931-0)
Supplement: Supplementary file 1 — Supplementary Information [file 41467_2023_36931_MOESM1_ESM.pdf]

SUPPLEMENTARY INFORMATION

Optical control of PIEZO1 channels

Authors

Francisco Andrés Peralta[1,2], Mélaine Balcon[1], Adeline Martz[1], Deniza Biljali[1], Federico Cevoli[1], Benoit Arnould[1], Antoine Taly[3,4], Thierry Chataigneau[1] and Thomas Grutter[1,2,*]

[1]Équipe de Chimie et Neurobiologie Moléculaire, Laboratoire de Conception et Application de Molécules Bioactives (CAMB) UMR 7199, Université de Strasbourg, Centre National de la Recherche Scientifique, Faculté de Pharmacie, 67401 Illkirch, France

[2]University of Strasbourg Institute for Advanced Studies (USIAS), 67000 Strasbourg, France

[3]Laboratoire de Biochimie Théorique, CNRS, Université Paris Cité, UPR 9080, France

[4]Institut de Biologie Physico-chimique, Fondation Edmond de Rothschild, France

[*]To whom correspondence should be addressed. (E-mail: grutter@unistra.fr).

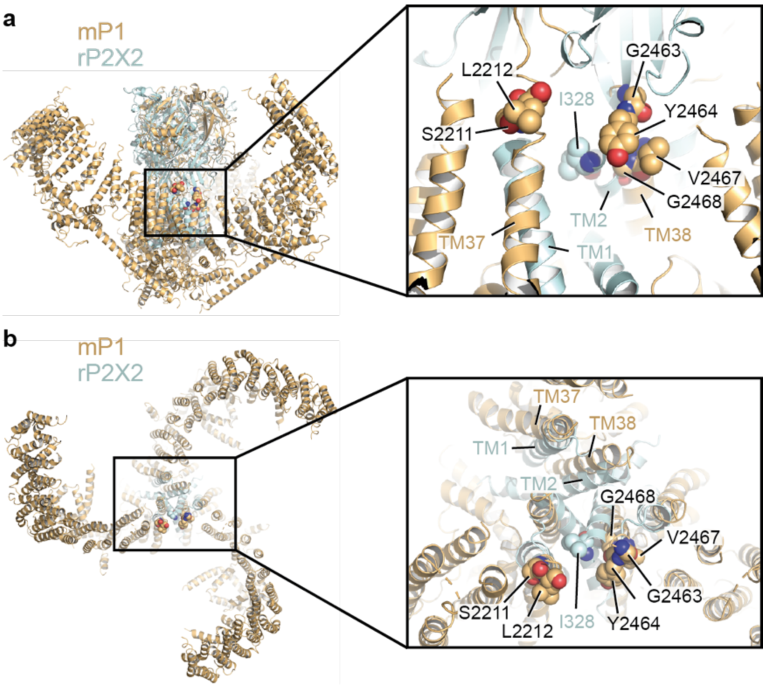

Supplementary Fig. 1. Structural alignment between mouse PIEZO1 central pore and rat P2X2 pore. Side (**a**) and top (**b**) views of rP2X2 model (pale blue ribbon) superimposed to mP1 structure (wheat ribbon) at the level of the pore-lining TM-helices (TM1 of rP2X2 to TM37 of mP1 and TM2 of rP2X2 to TM38 of mP1). Enlarged views of the extracellular region of the pore-lining TM-helices show I328 residue from rP2X2 (pale blue spheres) and selected mP1 residues (wheat spheres).

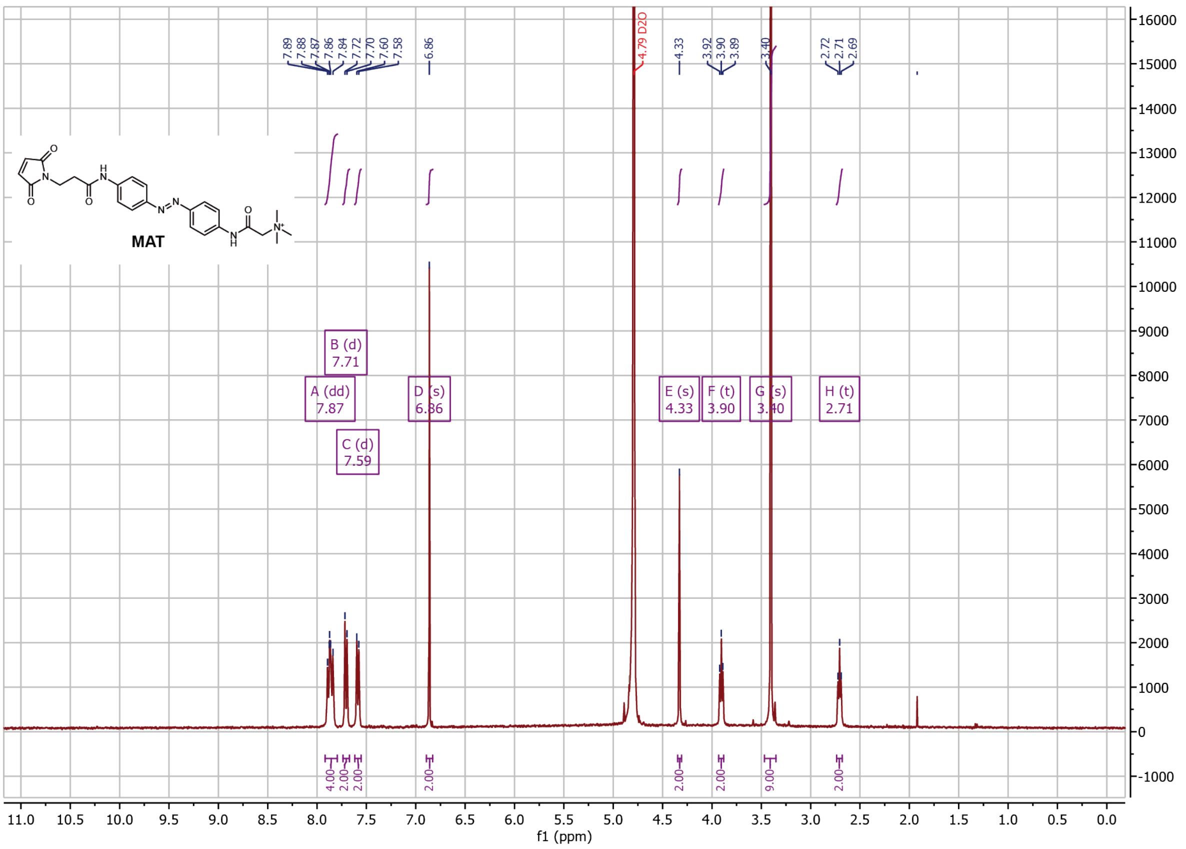

Supplementary Fig. 2. ^{1}H-NMR spectrum of compound MAT.

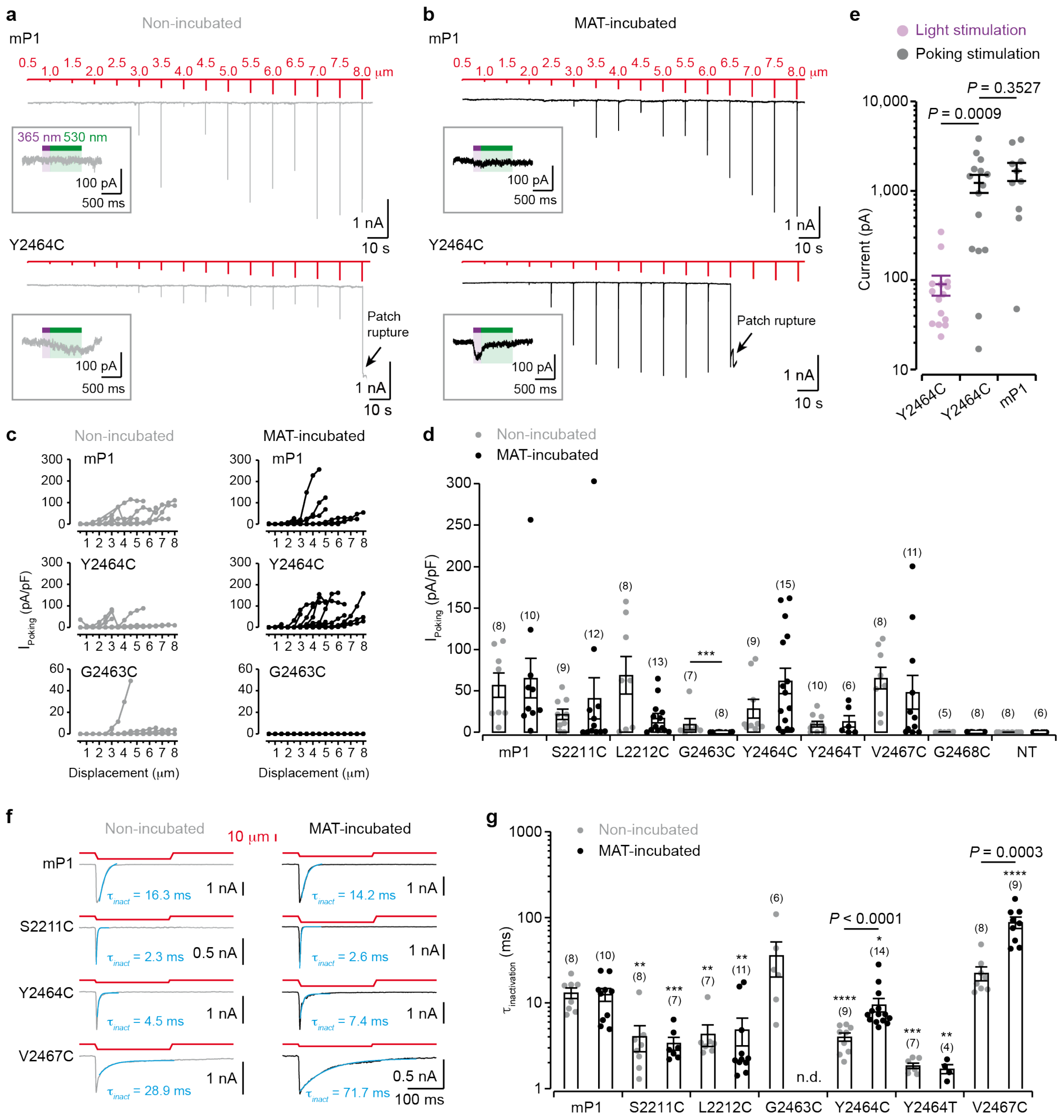

Supplementary Fig. 3. Functional characterization of mechanically-activated PIEZO1 constructs before and after MAT labeling. **a**, **b**, Full protocol partially shown in Figure 1d exhibiting a series of whole-cell inward currents evoked by poking of cells expressing mP1 or Y2464C mutant treated or not by MAT. Each cell was first irradiated (inset) and then transiently stimulated (duration 200 ms) by 16 downward mechanical steps of 0.5 µm at 1 µm ms^{-1}. Note that in some cases, whole-cell patches were lost before the end of the protocol in response to poking stimulations. **c**, Stimulus-response curves for each cell tested expressing the indicated constructs and incubated (right) or not (left) with MAT. **d**, Current density obtained from maximal poking-induced currents of all constructs incubated (black) or not (grey) with MAT (number of cells is indicated in parentheses). NT, non-transfected. Two-tailed Mann-Whitney test comparing data from non-incubated with MAT-incubated conditions: ***= 0.0003. **e**, Comparison of current amplitudes induced by light (violet) to those induced by the largest poking stimulations (grey) in the Y2464C mutant or mP1 incubated with MAT. Comparisons

with two-tailed Wilcoxon matched-pairs singed rank test for Y2464C data and two-tailed unpaired *t*-test for mP1 data (n = 15 cells for Y2464C light and poking stimulations and 10 cells for mP1 poking stimulation). **f**, Whole-cell inward currents evoked by poking in cells expressing the indicated constructs treated or not by MAT. Inactivated currents were fitted with an exponential equation giving τ values. **g**, Average τ values for each construct in cells treated (black) or not (grey) with MAT (number of cells is indicated in parentheses). n.d., not determined. Two-tailed Mann-Whitney test comparing to non-incubated control mP1 data. *P*-values (from left to right): **= 0.0019, ***= 0.0003, **= 0.0037, **= 0.005, ****< 0.0001, *= 0.0352, ***= 0.0003, **= 0.004, ****< 0.0001. Indicated *P*-values are from two-tailed Mann-Whitney test comparing data from non-incubated with MAT-incubated conditions. All data are presented as mean ± s.e.m.. Source data are provided as a Source Data file.

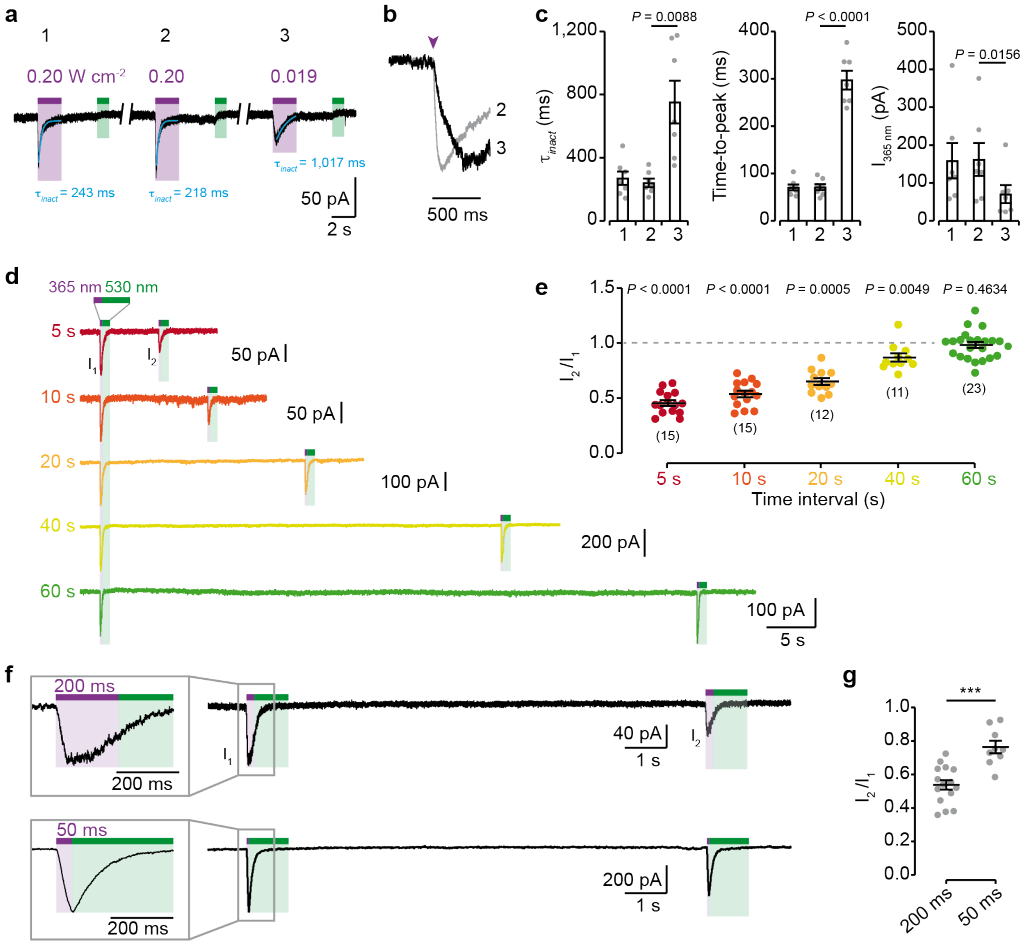

Supplementary Fig. 4. Functional characterization of light-induced inactivation and recovery of mOP1. **a**, Typical whole-cell currents at -80 mV recorded from the same cell expressing MAT-labeled Y2464C mutant (mOP1) in response to three successive cycles of 365-nm (violet) and 530-nm (green) at the indicated light intensity of the 365-nm LED. Inactivation currents were fitted with an exponential equation giving τ_{inact} values at 365 nm. **b**, Superimposed traces recorded from another cell of the second and third light-gated currents time-locked to light activation (arrow head). **c**, Average τ_{inact} (left), time-to-peak (middle) and current-density (right) values (n = 7 cells). Indicated *P*-values are from two-tailed paired *t*-test for τ_{inact} and time-to-peak data, and from two-tailed Wilcoxon matched-pairs signed rank test for current amplitude data. **d**, Typical traces of whole-cell inward currents evoked by two cycles (I_1 and I_2) of irradiation at 365-nm (200 ms) and 530-nm light (800 ms) separated by different time intervals of 5, 10, 20, 40 and 60 s. Each trace was recorded from a different cell. **e**, Average ratio (I_2/I_1) of currents induced at 365 nm in function of time interval (number of cells is indicated in parentheses). Indicated *P*-values are from two-tailed Wilcoxon test. **f**, Typical traces of whole-cell inward currents evoked by two cycles of irradiation at 365-nm (200 or 50 ms) and 530-nm light (800 or 950 ms) separated by a time interval of 10 s. Note the absence of current inactivation during the 50-ms irradiation at 365 nm. **g**, Average ratio (I_2/I_1) of currents in function of irradiation time at 365 nm (n = 15 cells for 200 ms and 9 cells for 50 ms). Two-tailed Mann-Whitney test, *P*-value ***= 0.0004. All data are presented as mean ± s.e.m.. Source data are provided as a Source Data file.

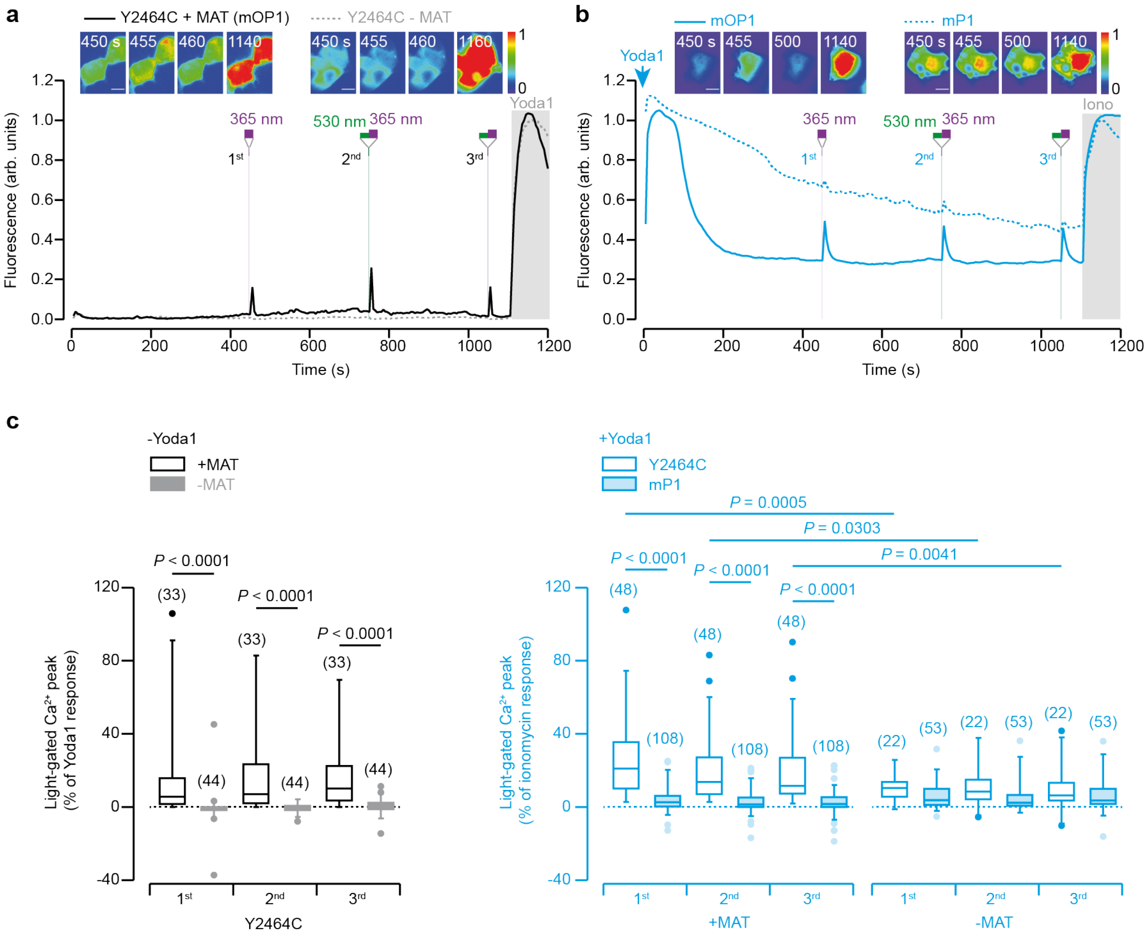

Supplementary Fig. 5. GCaMP6 Ca^{2+} imaging with mOP1. **a**, Time-lapse of fluorescence variation (arbitrary units) depicting intracellular Ca^{2+} dynamic in response to 1 s irradiations at 365-nm in cells expressing Y2464C treated (thick black trace) or nor (dotted grey trace) with MAT. Note 1-s pre-irradiations at 530 nm just before the second and third irradiation at 365 nm. Yoda1 (10 µM) was applied as control (grey shadow). Corresponding fluorescent images are shown on the top at the indicated times. Scale bar, 10 µm. **b**, Time-lapse of fluorescence variation (arbitrary units) depicting intracellular Ca^{2+} dynamic in the continuous presence of 10 µM Yoda1 (arrow, applied just before the onset of the experiments) in response to 1 s irradiations at 365-nm in MAT-treated cells expressing either mOP1 (thick cyan trace) or mP1 (dotted cyan trace). Ionomycin (5 µM) was applied as control (grey shadow). Corresponding fluorescent images are shown on the top at the indicated times. Scale bar, 10 µm. **c**, Box plot of light-gated Ca^{2+} peak responses normalized to that of ionomycin or Yoda1 in function of irradiation cycles in the absence or presence of Yoda1. Responses are presented as median (center), 25-75 percentile (box) and 5-95 percentile (whisker). Outliers extending beyond whiskers (1.25× whisker length) are shown. Indicated *P*-values are from two-tailed Mann-Whitney test. Number of cells is indicated in parentheses. Note the presence of unspecific, light-gated responses only in the presence of Yoda1 and in the absence of MAT (-MAT, untreated cells) expressing either Y2464C or mP1. Source data are provided as a Source Data file.

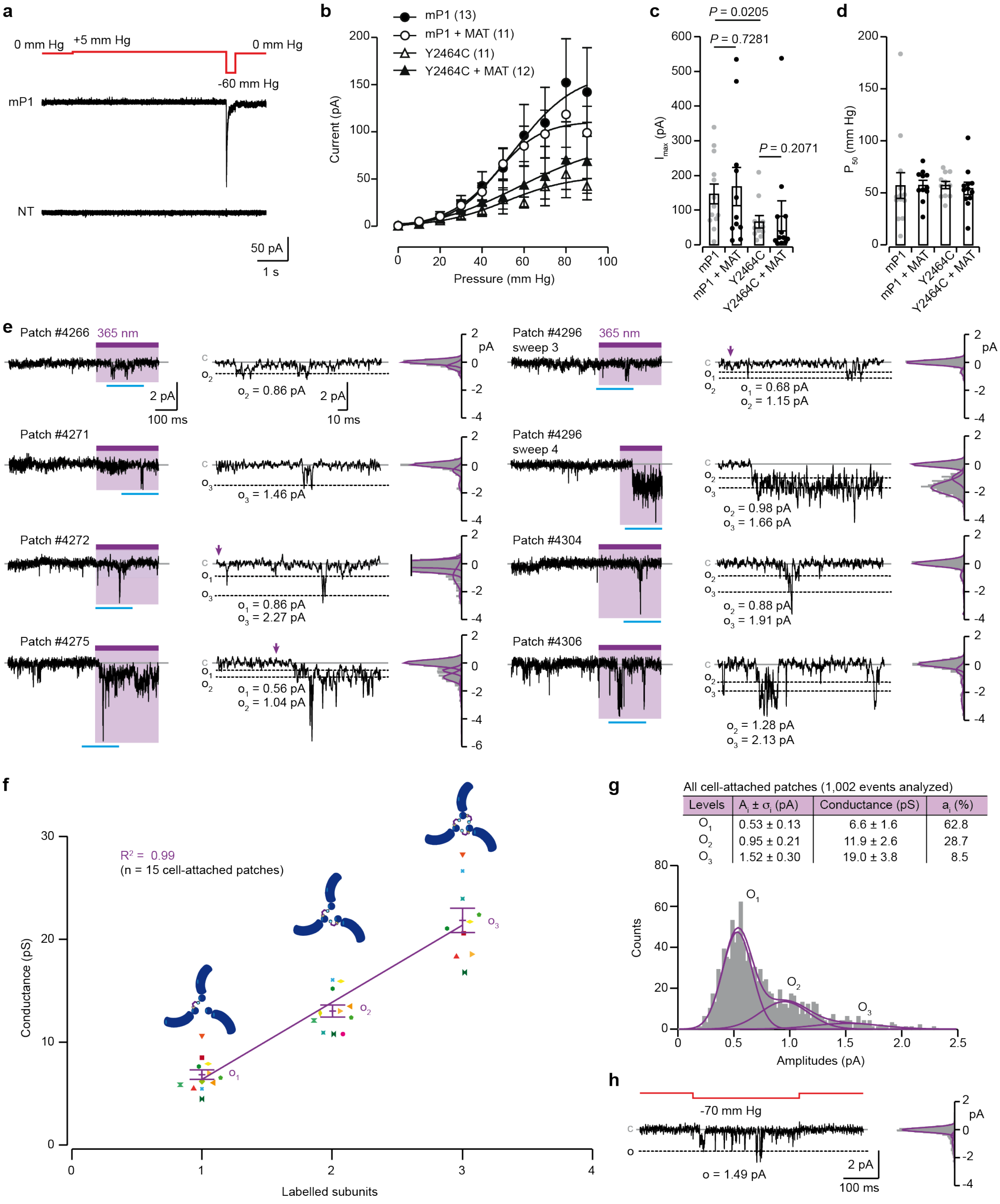

Supplementary Fig. 6. Functional characterization of pressure- and light-evoked responses in the cell-attached configuration. **a**, Typical inward currents in the cell-attached configuration evoked by applying negative pressure through the recording pipette (-60 mm Hg for 300 ms) at a holding potential of -80 mV. Shown are two patches from cells that were transfected (upper trace) or not (lower trace) with mP1. To minimize resting membrane tension, a pre-pulse of +5 mm Hg was applied to the patch for 5 s prior to mechanical stimulations, as previously described[1]. **b**, Current-pressure relationship of stretch-activated currents at – 80 mV from patches expressing the indicated construct treated or not with MAT. Data were fitted with a Boltzmann equation giving I_{max} and P_{50} (n = 11-13 patches, indicated in parentheses). **c**, **d**, Corresponding I_{max} and P_{50} values obtained from Boltzmann fittings. *P*-values are from two-

tailed Mann-Whitney test (n = 13 cells for mP1, 11 cells for mP1 + MAT, 11 cells for Y2464C and 12 cells for Y2464C + MAT). **e**, Left, examples of single-channel currents at -80mV in the cell-attached configuration elicited by light irradiation (365 nm and 530 nm) from MAT-treated cells expressing mOP1. Channel openings are downward deflections. Right, sections of the recordings indicated by cyan lines below traces are shown at a higher time resolution. Label c denotes closed channels and labels o_1, o_2 and o_3 represent the three open conductance levels. Violet arrows indicate the start of irradiations. Corresponding all-point histograms with Gaussian fits are shown right of the traces. Data were acquired at 40 kHz and filtered at 1 kHz. **f**, Light-induced conductance (at -80 mV) of o_1, o_2 and o_3 as a function of labeled subunits (n = 15 cell-attached patches). Each cell-attached patch is shown by a different symbol and color. Data were fitted with a linear regression (violet line) of the form $y = 7.5 \times x - 1.1$. Inset: cartoon depicting subunit labeling stoichiometry with one, two and three MAT molecules putatively corresponding, respectively, to o_1, o_2 and o_3. All data are presented as mean ± s.e.m.. **g**, Distribution of detected events obtained from all cell-attached patches (n = 15) as a function of current amplitude. Amplitude histogram was fitted with three Gaussians and fitted values are shown on the top. Note the presence of three conductance level values that were very similar to those determined individually from each patch. **h**, Single-channel currents at -80 mV in the cell-attached configuration elicited by pressure from cells expressing Y2464C (not treated with MAT). Channel openings are downward deflections. Labels c and o denote, respectively, closed and open conductance levels. Corresponding all-point histogram with Gaussian fits is shown right of the trace. Data were acquired at 20 kHz and filtered at 1 kHz. Source data are provided as a Source Data file.

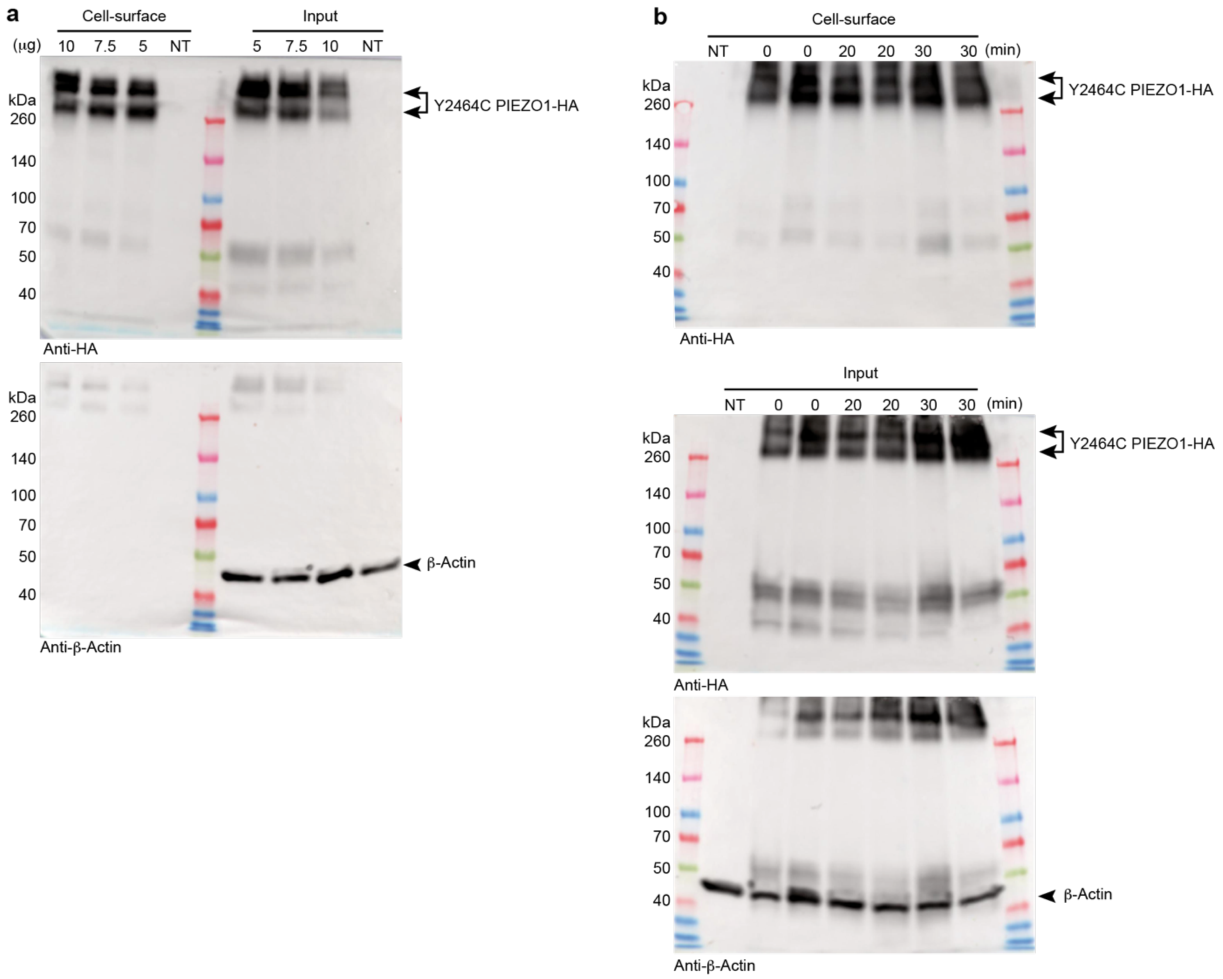

Supplementary Fig. 7. Cell-surface protein biotinylation assay of Y2464C PIEZO1-HA. a, Uncropped Western blot of biotinylated membrane-associated proteins (cell-surface) or total cell lysate (input) separated by SDS-PAGE from HEK-P1KO cells transiently transfected with 5, 7.5 or 10 µg of plasmid encoding hemagglutinin (HA) tagged Y2464C mPIEZO1 (Y2464C PIEZO1-HA). The blot was probed with anti-HA antibody. Note the presence of specific bands running at the expected molecular masses (~300 kDa), as previously described[2,3]. Below is shown the same blot re-probed with anti-β-Actin. **b**, Uncropped Western blots of biotinylated membrane-associated proteins (cell-surface) or total cell lysate (input) separated by SDS-PAGE from HEK-P1KO cells transiently transfected with 5 µg of plasmid encoding Y2464C PIEZO1-HA. Before biotinylation, transfected cells were incubated with 200 µM MAT for 20 or 30 min (n = 2 independent replicates). Blots were probed with anti-HA antibody. Below input is shown the same blot re-probed with anti-β-Actin. Apparent molecular weights are indicated on the left of Western blots. NT, non-transfected.

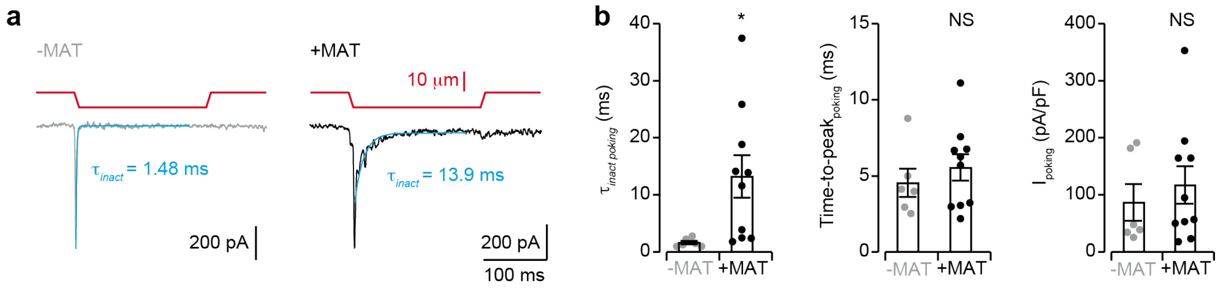

Supplementary Fig. 8. Poking-evoked currents in R1762C/E2257C/Y2464C in the presence of DTT. a, Typical whole-cell currents recorded at -80 mV elicited by cell poking in cells treated or not by MAT. DTT (10 mM) was bath applied at least 3 minutes before recordings. Inactivated currents were fitted with an exponential equation giving τ values. **b**, Average τ, time-to-peak and current density values in the absence (grey) or presence (black) of MAT (n = 6 cells without MAT and 10 cells with MAT). Two-tailed unpaired t test (for τ_{inact} and time-to-peak) or two-tailed Mann-Whitney test (current density). *P*-values: *= 0.0314, NS = 0.4663 and 0.5622 (from left to right). NS, not significant. All data are presented as mean ± s.e.m.. Source data are provided as a Source Data file.

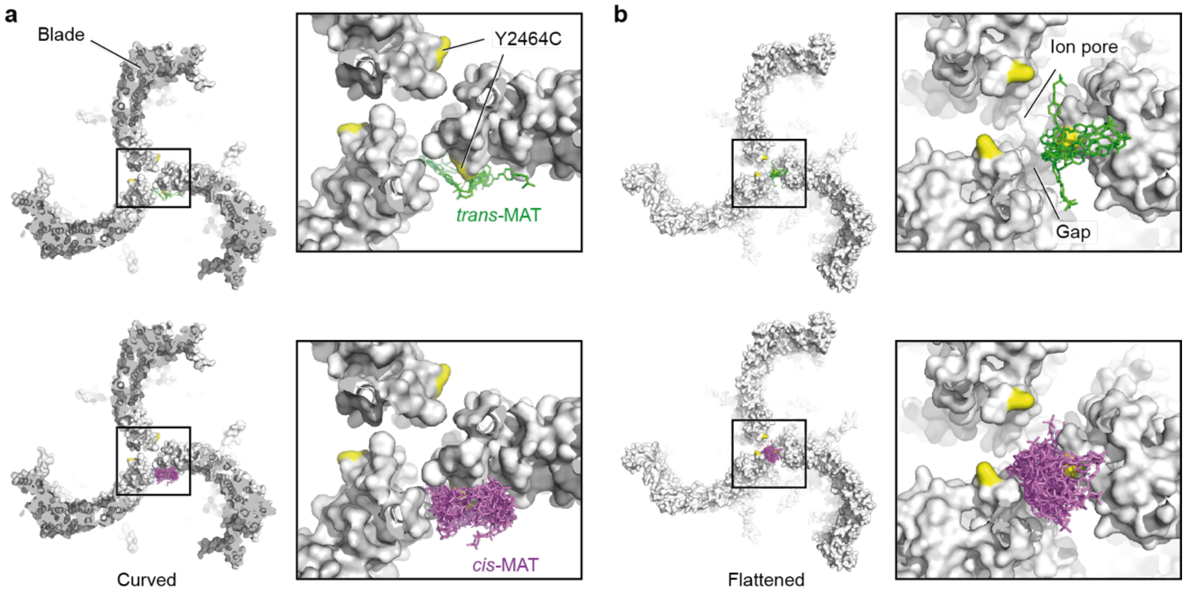

Supplementary Fig. 9. Molecular docking of *trans*-MAT and *cis*-MAT in mP1 structures. **a**, **b**, Top views of space-filling models of the Y2464C mutant modelled from the cryo-EM mP1 structures solved in a curved or flattened state[4]. Docking poses of *trans*-MAT (top) and *cis*-MAT (bottom) are shown for each state and color-coded according to irradiation wavelengths. Insert, enlarged views with the position of the Y2464C mutation (yellow). Note the presence of a gap in the flattened model that was not filled with *trans*-MAT poses.

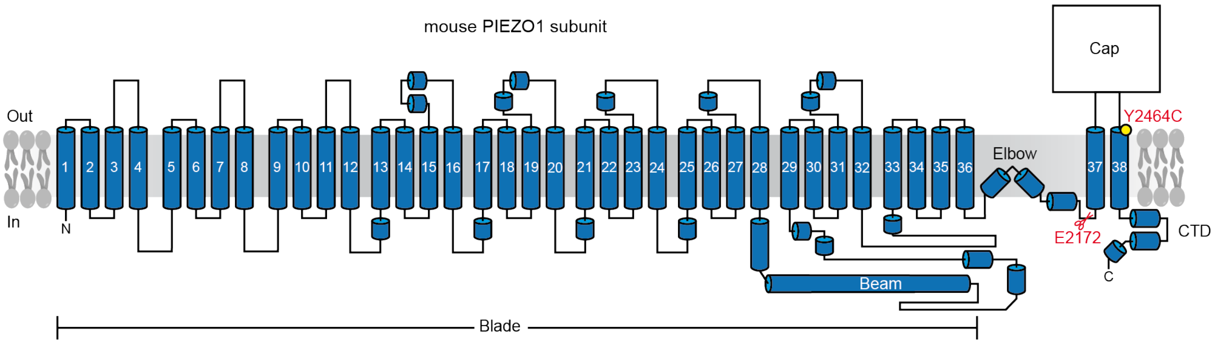

Supplementary Fig. 10. Topology model of the full-length mP1 subunit. The Δblade mOP1 started at residue E2172 and contained the selected Y2464C mutation. Also shown are the localization of TM helices and PIEZO structural features. CDT, C-terminal domain.

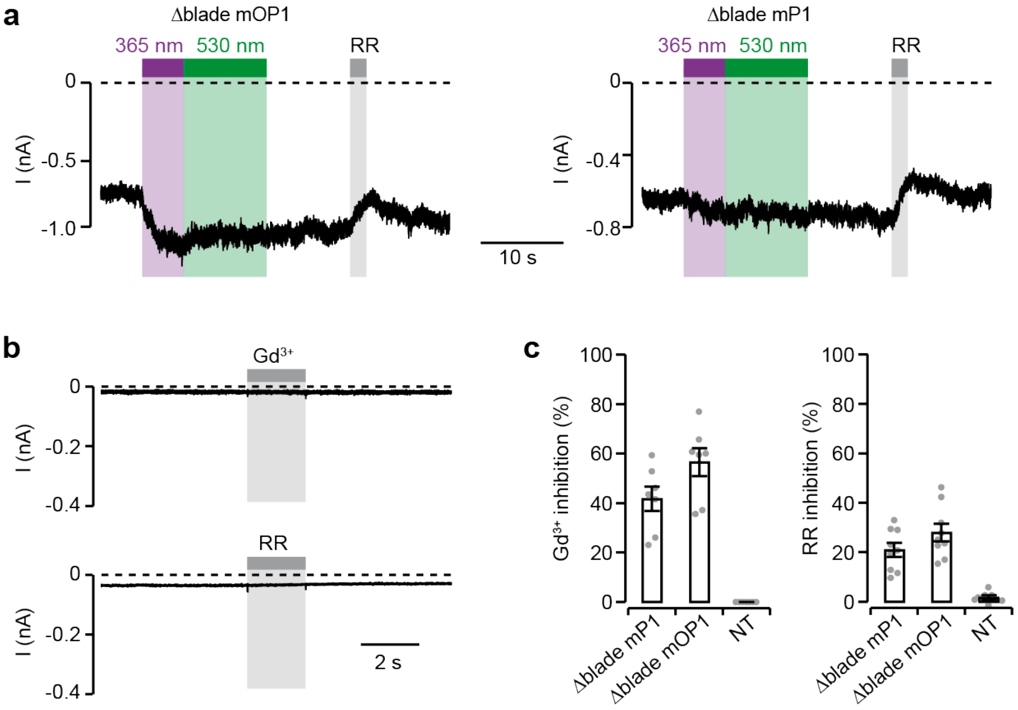

Supplementary Fig. 11. Specific reduction of holding currents upon Gd^{3+} or RR application in cells expressing truncated PIEZO1 channels. **a**. Representative whole-cell currents at -80 mV of Δblade mOP1 (left) and Δblade mP1 (right) in response to light irradiation and 30 µM RR application from cells treated with MAT. **b**, Representative whole-cell currents from non-transfected cells recorded during a 30-µM Gd^{3+} (top) or 30-µM RR (bottom) application. Note the absence of leaky currents. **c**, Average Gd^{3+}- and RR-induced inhibitions of holding currents recorded in cells transfected with Δblade mP1 or Δblade mOP1 (mean ± s.e.m., n = 7 cells for Gd^{3+} inhibition, 9 cells for RR inhibition and 7 cells for NT). NT, non-transfected cells, RR, ruthenium red. Source data are provided as a Source Data file.

Supplementary References

1 Lewis, A. H. & Grandl, J. Mechanical sensitivity of Piezo1 ion channels can be tuned by cellular membrane tension. *Elife* **4**, doi:10.7554/eLife.12088 (2015).

2 Ranade, S. S. *et al.* Piezo1, a mechanically activated ion channel, is required for vascular development in mice. *Proc Natl Acad Sci U S A* **111**, 10347-10352, doi:10.1073/pnas.1409233111 (2014).

3 Li, J. V. *et al.* Modified N-linked glycosylation status predicts trafficking defective human Piezo1 channel mutations. *Commun Biol* **4**, 1038, doi:10.1038/s42003-021-02528-w (2021).

4 Yang, X. *et al.* Structure deformation and curvature sensing of PIEZO1 in lipid membranes. *Nature*, doi:10.1038/s41586-022-04574-8 (2022).
