# Supplementary material for: Optical control of PIEZO1 channels
Source: Nat Commun. 2023 Mar 7;14:1269. doi: 10.1038/s41467-023-36931-0 (PMC9992513; doi:10.1038/s41467-023-36931-0)
Supplement: Supplementary file 4 — Reporting Summary [file 41467_2023_36931_MOESM4_ESM.pdf]

nature portfolio

Corresponding author(s): Pr. Thomas Grutter

Last updated by author(s): Feb 22, 2023

Reporting Summary

Nature Portfolio wishes to improve the reproducibility of the work that we publish. This form provides structure for consistency and transparency in reporting. For further information on Nature Portfolio policies, see our Editorial Policies and the Editorial Policy Checklist.

Statistics

For all statistical analyses, confirm that the following items are present in the figure legend, table legend, main text, or Methods section.

n/a	Confirmed	
[ ]	[x]	The exact sample size (n) for each experimental group/condition, given as a discrete number and unit of measurement
[ ]	[x]	A statement on whether measurements were taken from distinct samples or whether the same sample was measured repeatedly
[ ]	[x]	The statistical test(s) used AND whether they are one- or two-sided<br>*Only common tests should be described solely by name; describe more complex techniques in the Methods section.*
[x]	[ ]	A description of all covariates tested
[ ]	[x]	A description of any assumptions or corrections, such as tests of normality and adjustment for multiple comparisons
[ ]	[x]	A full description of the statistical parameters including central tendency (e.g. means) or other basic estimates (e.g. regression coefficient) AND variation (e.g. standard deviation) or associated estimates of uncertainty (e.g. confidence intervals)
[ ]	[x]	For null hypothesis testing, the test statistic (e.g. F, t, r) with confidence intervals, effect sizes, degrees of freedom and P value noted<br>*Give P values as exact values whenever suitable.*
[x]	[ ]	For Bayesian analysis, information on the choice of priors and Markov chain Monte Carlo settings
[ ]	[x]	For hierarchical and complex designs, identification of the appropriate level for tests and full reporting of outcomes
[x]	[ ]	Estimates of effect sizes (e.g. Cohen's d, Pearson's r), indicating how they were calculated

Our web collection on statistics for biologists contains articles on many of the points above.

Software and code

Policy information about availability of computer code

Data collection	Electrophysiology: PATCHMASTER 2x91<br>Calcium imaging: Live Acquisition Software 2.6.0.29
Data analysis	Electrophysiology: Fitmaster 2x73<br>Electrophysiology: Igor Pro 6.36<br>Electrophysiology: TAC 3.0<br>Electrophysiology: TACFit 3.0<br>Calcium imaging: ImageJ 1.53a<br>Electrophysiology and calcium imaging: GraphPad Prism 9<br>Modelling: PyMol 2.5.4<br>Modelling: Scwrl4<br>Modelling: smina (based on AutoDock Vina 1.1.2)<br>Modelling: Open Babel 2.4.1<br>Chemical synthesis: MestReNova 14.2.3-29241

For manuscripts utilizing custom algorithms or software that are central to the research but not yet described in published literature, software must be made available to editors and reviewers. We strongly encourage code deposition in a community repository (e.g. GitHub). See the Nature Portfolio guidelines for submitting code & software for further information.

Data

Policy information about availability of data

All manuscripts must include a data availability statement. This statement should provide the following information, where applicable:

- Accession codes, unique identifiers, or web links for publicly available datasets
- A description of any restrictions on data availability
- For clinical datasets or third party data, please ensure that the statement adheres to our policy

All data generated in this study are provided in the main text and Supplementary Information. Previously published structures from the PDB can be accessed via accession codes: 5Z10, 7WLT, 7WLU.

Human research participants

Policy information about studies involving human research participants and Sex and Gender in Research.

Reporting on sex and gender	N/A
Population characteristics	N/A
Recruitment	N/A
Ethics oversight	N/A

Note that full information on the approval of the study protocol must also be provided in the manuscript.

Field-specific reporting

Please select the one below that is the best fit for your research. If you are not sure, read the appropriate sections before making your selection.

[x] Life sciences [ ] Behavioural & social sciences [ ] Ecological, evolutionary & environmental sciences

For a reference copy of the document with all sections, see nature.com/documents/nr-reporting-summary-flat.pdf

Life sciences study design

All studies must disclose on these points even when the disclosure is negative.

Sample size	No sample size was calculated. However, based on prior studies that used similar methodology (electrophysiology and calcium imaging), we considered that 4-25 cells are sufficient to obtain reliable results for whole-cell experiments, that 6-15 patches are sufficient to determine single channel conductance, and that 22-108 cells are sufficient to quantify intracellular calcium responses.
Data exclusions	For electrophysiological experiments, we excluded from the analyses cells that displayed excessive or unstable leak currents (e.g. more than 500 pA for whole-cell recordings). For patch-clamp DTT experiments, we excluded from the analyses cells that displayed unstable baseline currents due to irregular DTT perfusion. For single channel recordings, we excluded patches that contained no conductance. For calcium imaging, we excluded cells that did not respond to the Yoda-1 or ionomycin control or cells for which basal fluorescence (in the absence of Yoda-1) was high.
Replication	Experiments were repeated several times (at least 3 times for each transfection), over a minimum of 2 independent transfections. Where relevant, the number of cells/patches analysed is indicated in the figures or legends. All experimental replications were successful.
Randomization	Randomization is not relevant to this study, as samples are not required to be allocated into experimental groups. No animals or human research participants are involved in this study.
Blinding	Blinding is not relevant to this study, as samples are not required to be allocated into experimental groups. No animals or human research participants are involved in this study.

Reporting for specific materials, systems and methods

We require information from authors about some types of materials, experimental systems and methods used in many studies. Here, indicate whether each material, system or method listed is relevant to your study. If you are not sure if a list item applies to your research, read the appropriate section before selecting a response.

Materials & experimental systems

n/a	Involved in the study	
☐	☒	Antibodies
☐	☒	Eukaryotic cell lines
☒	☐	Palaeontology and archaeology
☒	☐	Animals and other organisms
☒	☐	Clinical data
☒	☐	Dual use research of concern

Methods

n/a	Involved in the study	
☒	☐	ChIP-seq
☒	☐	Flow cytometry
☒	☐	MRI-based neuroimaging

Antibodies

Antibodies used

Primary antibody: Mouse monoclonal anti-HA (Invitrogen 26183) diluted 1:500. Clone: 2-2.2.14
Secondary antibody: Goat anti-Mouse HRP (Invitrogen 31430) diluted 1:10,000.

Validation

Antibody specificity was validated by performing "non-transfected" controls, in which no signal was detected (Supplementary Fig. 7).
Mouse monoclonal anti-HA: (from manufacturer's website) "26183 is specific for the HA peptide YPYDVPDYA. The antibody has been used successfully in Western blot and immunoprecipitation applications."
Goat anti-Mouse HRP: (from manufacturer's website) "Product # 31430 has been successfully used in Western blot, IHC and IP applications".

Eukaryotic cell lines

Policy information about cell lines and Sex and Gender in Research

Cell line source(s)

HEK-P1KO cell line was a gift from Dr. Ardem Patapoutian and Dr. Eric Honoré.

Authentication

HEK-P1KO cell line was not authenticated.

Mycoplasma contamination

HEK-P1KO cell line was tested negative for mycoplasma contamination.

Commonly misidentified lines
(See ICLAC register)

No commonly misidentified cell lines were used.
