# Supplementary material for: Optical control of PIEZO1 channels
Source: Nat Commun. 2023 Mar 7;14:1269. doi: 10.1038/s41467-023-36931-0 (PMC9992513; doi:10.1038/s41467-023-36931-0)
Supplement: Supplementary file 5 — Description of Additional Supplementary Files [file 41467_2023_36931_MOESM5_ESM.pdf]

Title: Supplementary Data 1
Description: List of primers used.
